# Behavioural interventions to address rational use of antibiotics in outpatient settings of low‐income and lower‐middle‐income countries

**DOI:** 10.1111/tmi.13550

**Published:** 2021-01-28

**Authors:** Mohit M. Nair, Raman Mahajan, Sakib Burza, Maurice P. Zeegers

**Affiliations:** ^1^ Nutrition and Translational Research in Metabolism, Care and Public Health Research Institute Maastricht University Maastricht The Netherlands; ^2^ Médecins Sans Frontières New Delhi India

**Keywords:** antibiotic resistance, systematic review, behavioural

## Abstract

**Objectives:**

To explore the current evidence on interventions to influence antibiotic prescribing behaviour of health professionals in outpatient settings in low‐income and lower‐middle‐income countries, an underrepresented area in the literature.

**Methods:**

The systematic review protocol for this study was registered in PROSPERO (CRD42020170504). We searched PubMed, Embase and the Cochrane Central Register of Controlled Trials (CENTRAL) for studies relating to antibiotic prescribing of health professionals in outpatient settings in low‐income and lower‐middle‐income countries. Behavioural interventions were classified as persuasive, enabling, restrictive, structural or bundle (mix of different interventions). In total, 3,514 abstracts were screened and 42 studies were selected for full‐text review, with 13 studies included in the final narrative synthesis.

**Results:**

Of the 13 included studies, five were conducted in Vietnam, two in Sudan, two in Tanzania, two in India and two in Kenya. All studies were conducted in the outpatient or ambulatory setting: eight took place in primary health centres, two in private clinics and three in pharmacies. Our review found that enabling or educational interventions alone may not be sufficient to overcome the ingrained incentives to link revenue generation to sales of antibiotics, and hence, their inappropriate prescription or misuse. Bundle interventions appear to be very effective at changing prescription behaviour among healthcare providers, including drug sellers and pharmacists.

**Conclusions:**

Multi‐faceted bundle interventions that combine regulation enforcement with face‐to‐face education and peer influence may be more effective than educational interventions alone at curbing inappropriate antibiotic use.

## Introduction

The advent of antibiotics has ushered in an era of increased life expectancy and reduced mortality and morbidity from infectious diseases [1]. However, overuse of antibiotics and irrational prescriptions of antibiotics, especially in developing or emerging economies, has limited the effectiveness of antibiotics by contributing to antibiotic resistance (ABR), a phenomenon wherein bacteria develop the ability to withstand or resist the effects of antibiotics and are uninhibited by them [2]. One multi‐country study from low‐income and lower‐middle‐income countries (LMICs) found that children received an average of 25 antibiotic prescriptions in the first five years of life [2]. Given the high rates of antibiotic overuse and misuse, WHO warned in 2014 that ABR poses a serious threat in every region [3].

Drivers of antibiotic resistance have been investigated globally [4, 5, 6, 7, 8, 9, 10, 11, 12, 13], but data analysing antibiotic stewardship or other behavioural interventions to address prescription behaviour of healthcare providers in outpatient settings as drivers of antibiotic use are very scarce [14, 15, 16, 17, 18, 19, 20]. Some of these reviews have either been overly broad in scope with a predominant focus on context over behavioural interventions, or they failed to include evidence from informal and private sector settings within healthcare in LMICs. Healthcare providers, especially in low‐income or other limited‐resource settings, can be formally trained (e.g. medical doctors, nurses and pharmacists) or informal practitioners (untrained medical practitioners or practitioners of alternative medicine). In India, for example there is a government ministry for Ayurveda, Yoga & Naturopathy, Unani, Siddha and Homeopathy (AYUSH) that is dedicated to training and providing alternative systems of medical care [21]. The focus on outpatient care is particularly important in low‐income settings, where the relation between overwhelmed government healthcare systems and burgeoning private, informal providers is poorly understood in the context of antibiotic consumption [1].

Most of the evidence comes from developed countries and involves more complex multi‐faceted strategies such as electronic decision support, electronic health record prompts and automated peer comparison interventions [14, 18, 19, 22]. However, it is unclear whether similar interventions are as effective or could be applicable in LMICs. Such contexts often face a high burden of communicable diseases, which are not always amenable to expensive or high‐tech interventions involving electronic health records [2, 19].

To that end, our systematic review explores the current evidence on behavioural interventions influencing antibiotic prescribing behaviour of health professionals in outpatient settings in LMICs.

## Methods

The systematic review protocol for this study was registered in PROSPERO (CRD42020170504). We searched PubMed, Embase and the Cochrane Central Register of Controlled Trials (CENTRAL) for studies relating to antibiotic prescribing of health professionals in outpatient settings in LMICs, including bibliographies of retrieved articles. Only articles published in English between 2001 and 2019 were included in the review. Articles published before 2001 were excluded as this was when the first global plans addressing antibiotic resistance emerged, and the objective of the systematic review was to examine the latest evidence on behavioural interventions to address antibiotic resistance [23].

Behavioural interventions were defined and classified as persuasive (prescription audits and feedback advice), enabling (education or guidelines on antibiotic use), restrictive (expert approval prior to using certain antibiotics), structural (introduction of a new diagnostic test or clinical algorithm to guide prescriptions) or bundle (mix of different interventions), based on a prior systematic review by Davey et al using the EPOC taxonomy [14, 24]. Randomised controlled trials, non‐randomised controlled trials, time series, uncontrolled before‐and‐after studies and qualitative studies were included if they reported on outcomes related to knowledge, attitudes and practices regarding antibiotic use following an intervention among prescribers. Interventions focusing on microbiological tests, improving infection prevention and control, or hospital inpatients and nursing homes were excluded. Interventions with retailers, including pharmacies, drug stores or informal sellers, were also included. Studies that did not have full‐text articles available were excluded if the original authors were not contactable. The focus was on outpatient or ambulatory care in LMICs, according to World Bank classification [25]. Studies focusing on malaria, HIV, malnutrition or other infectious diseases without directly dealing with antibiotic stewardship were excluded.

**Figure 1:**
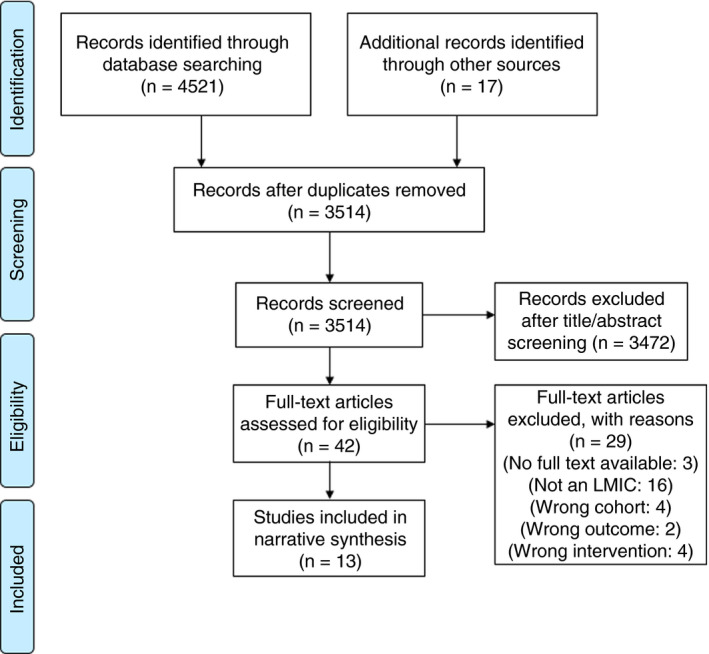
PRISMA diagram.[Colour figure can be viewed at wileyonlinelibrary.com]

Search results from PubMed, Embase and CENTRAL were imported into Excel with 4,521 entries in total. After removing duplicates, 3,514 remained. MN conducted a detailed title and abstract screen, and RM subsequently reviewed all titles and abstracts to identify interventions addressing antibiotic prescription behaviour (see PRISMA diagram in Figure 1 below). Study data were extracted and study quality was assessed independently by two authors. In order to assess the risk of bias in the selected studies, two researchers independently applied the Cochrane Risk of Bias criteria to indicate whether each study posed a high, low or unclear risk of bias. Both reviewers discussed the studies after an independent analysis and reached consensus regarding the criteria.

Forty‐two studies were identified for full‐text review after an initial title and abstract screening. A pre‐designed data extraction sheet was used to include information on study design, type of intervention, type of targeted behaviour, participants, setting, methods, outcomes and results to extract results. Two reviewers independently conducted full‐text reviews of all 42 studies and excluded 29. The majority of these 29 studies were excluded due to misclassification as an LMIC: for instance, several studies were from China, which the World Bank currently classifies as an upper‐middle‐income country. 13 studies were included after full‐text review [26, 27, 28, 29, 30, 31, 32, 33, 34, 35, 36, 37, 38]. Given the heterogeneity in interventions and study designs, we present our results using a narrative synthesis approach without a meta‐analysis. In keeping with a narrative synthesis approach to systematic reviews, a qualitative and textual approach was adopted to compare, contrast and summarise the main themes from included studies rather than relying on statistical data through meta‐analysis. This ensured that the overarching ‘story’ from each of the studies was presented beyond limiting the results to the effectiveness of a particular intervention.

## Results

Out of the 13 included studies, five were conducted in Vietnam (one of these studies took place in both Vietnam and Thailand), two in Sudan, two in Tanzania, two in India and two in Kenya. All studies were conducted in the outpatient or ambulatory setting: eight took place in primary health centres (PHCs), two in private clinics and three in pharmacies. Four studies involved public facilities, eight involved private facilities and one study incorporated a mix of public and private facilities. Table 1 details characteristics of included studies.

**Table 1:** Characteristics of studies examining behavioural interventions to address antibiotic resistance in ambulatory settings in low‐income or lower‐middle‐income countries

Authors	Type of study	Type of intervention	Description of intervention	Duration of intervention	Country	Year	Type of setting	Private or public sector	Outcome(s)	Results
Kleczka et al [26]	Pre‐post design	Bundle	Rubber stamp templates, clinical practice guidelines, low‐budget smartphone and one continuing medical education session per month	6 months	Kenya	2019	Outpatient: primary healthcare clinics	Private	Antibiotic prescription rates ‘Ideal’ vs ‘actual’ antibiotic use Completeness of documentation Appropriateness of diagnosis and management	Antibiotic prescription rates > 90% for four infectious diseases Intervention did not affect # of antibiotics, but use of nitrofurantonin (appropriate, narrow‐spectrum antibiotic) increased from 9.2% to 29.9% (*P* < 0.0001) Use of broad‐spectrum quinolones decreased from 30% to 16.1% (*P* < 0.05)
Das et al [27]	Randomised controlled trial	Enabling	72 sessions and 150 teaching hours for informal providers over a 9‐month period, covering medical conditions, triage and avoidance of harmful practices.	9 months	India	2016	Outpatient: private clinics with informal providers	Private	Correct case management Use of unnecessary medicines and antibiotics	Improvement in correct case management (7.9%; *P* < 0.05) No statistically significant effect on use of unnecessary medicines and antibiotics
Lunn AD [28]	Pre‐post design	Bundle	Multi‐faceted intervention: repeated process of audit and feedback, interactive training sessions, one‐to‐one case‐based discussion, antibiotic guideline development and coding updates	4 months	India	2018	Outpatient: non‐governmental organisation’s primary health centre clinics	Primary care clinics (private non‐governmental organisation)	Antibiotic prescription rates	Dramatic decrease in antibiotic prescriptions from 62.6% to 7.2% (no *P*‐value provided).
Hoa et al [29]	Randomised controlled trial	Enabling	Six training sessions on acute respiratory infection management, six training sessions on acute respiratory infection case scenario management and poster distribution	7 months	Vietnam	2017	Outpatient: public and private personnel	Mixed	Difference in improvement of knowledge Difference in improvement of practice	Mean total knowledge score improved in the intervention group by 1.17 points (*P* < 0.001) 28% reduction in antibiotic use (*P* < 0.001) for mild acute respiratory infections
Korom et al [30]	Pre‐post design	Bundle	Clinical guideline for management of uncomplicated urinary tract infection + peer‐to‐peer review of documentation + discussion of a recently published, peer‐reviewed Kenyan study describing local AMR patterns	12 months	Kenya	2017	Outpatient: two semi‐urban primary health centres	Private	Appropriate antibiotic prescription Composite quality score	Appropriate antibiotic prescription improved from 19% to 68% (*P* < 0.001) Modest improvement in composite quality scores from an average of 2.16 to 3.00 on a 5‐point scale (*P* < 0.0001)
Rambaud‐Althaus et al [31]	Cluster randomised controlled trial	Structural	Paper algorithm + electronic algorithm on a smartphone + control	4 months	Tanzania	2017	Outpatient: 9 primary health centres (3 health centres and 6 dispensaries)	Public	Proportion of children checked for danger signs Antibiotic prescription rate	Use of smartphones vs. paper was associated with a significant increase in children checked for danger signs from 41% to 74% (*P* = 0.04) Antibiotic prescription rates decreased from 70% in the control arm to 26% in the paper arm and 25% in the electronic arm (*P* < 0.001)
Eltayeb et al [32]	Pre‐post design	Bundle	Audit and feedback + audit and feedback along with guidelines and two seminars + audit and feedback along with academic detailing and a copy of guidelines	2 months	Sudan	2005	Outpatient: 20 health centres	Public	Number of inappropriate prescriptions according to diagnosis Number of prescriptions with inappropriate dose and/or duration of therapy	Audit and feedback, academic detailing and practice guidelines together reduced the number of inappropriate prescriptions by 50% (*P* < 0.001) Audit and feedback alone reduced prescriptions by 16% (*P* = 0.127)
Shao et al [33]	Controlled, non‐inferiority trial	Structural	Algorithm for management of childhood illness available electronically and on paper + face‐to‐face supervision	1 month	Tanzania	2015	Outpatient: 4 PHCs	Private	Cure rate for children at day 7 and 14 Proportion of children who received antibiotics	97.3% were cured on day 7 in the intervention arm compared to 92% in the control arm (*P* < 0.001). 15.4% were prescribed antibiotics in the intervention arm compared to 84.3% in the control arm (*P* < 0.001)
Awad et al [34]	Pre‐post design	Bundle	No intervention (control); 2) audit and feedback; 3) audit and feedback + seminar; 4) audit and feedback + academic detailing	4 months	Sudan	2006	Outpatient: 20 health centres	Public	Total number of encounters with antibiotics prescribed	Audit and feedback along with academic detailing reduced the mean number of encounters with a prescribed antibiotic by 6.3 1‐month post‐intervention and 7.7 3‐months post‐intervention in comparison to the control group (*P* < 0.001) Audit and feedback along with seminars and audit and feedback alone reduced mean number of encounters with a prescribed antibiotic by 5.3 1‐month post‐intervention (*P* < 0.001) and 1.4 1‐month post‐intervention (*P* = 0.121).
Chalker et al [35]	Randomised controlled trial	Bundle	Enforcement of regulations with local inspectors + education + peer review	3 months	Vietnam and Thailand	2005	Outpatient: 68 pharmacies in Hanoi and 78 pharmacies in Bangkok	Private	Dispensing of steroids and antibiotics as requested Proportion of dispensers who asked for a prescription prior to dispensing antibiotics Proportion of dispensers who asked no questions or gave no advice prior to dispensing antibiotics	Dispensing of antibiotics improved in Hanoi after peer review intervention from 95% to 71% (*P* = 0.0125); smaller improvement post educational intervention from 90% to 69% (*P* = 0.0471); no statistically significant difference between intervention and control arms in Bangkok Number of staff who asked for prescriptions increased in the intervention group post education intervention (19% vs. 5%; *P* = 0.0127) and post peer review intervention (18% vs. 5%; *P* = 0.0075) compared to control groups.
Chuc et al [36]	Cluster randomised controlled trial with time series design	Bundle	Three interventions were applied sequentially: regulatory enforcement, education and peer influence	3 months	Vietnam	2002	Outpatient: 68 pharmacies	Private	Dispensing of antibiotics for acute respiratory infections Dispensing of correct syndromic treatment for sexually transmitted diseases Dispensing of prednisolone Dispensing of cefalexin	Dispensing of antibiotics decreased in intervention pharmacies from 45% to 30% (*P* < 0.02) and increased in control pharmacies from 39% to 42% (*P* < 0.05) Correct syndromic treatment for sexually transmitted diseases increased more in intervention pharmacies (3 to 30%; *P* < 0.05) compared to control pharmacies (4 to 19%; *P* < 0.05) Dispensing of cefalexin decreased from 95% to 56% in intervention pharmacies compared to 94% to 89% in control pharmacies (*P* < 0.01). Fewer steroids were dispensed in the intervention arm (78% to 17%) compared to the control arm (73% to 58%; *P* < 0.01).
Chalker et al [37]	Randomised controlled trial	Bundle	Regulations enforcement, face‐to‐face education and peer influence.	17 months	Vietnam	2002	Outpatient: 22 matched pair intervention and control pharmacies	Private	Change in knowledge Reported change in practice for correct management of tracer conditions	For acute respiratory infections, more drug sellers reported they would ask questions regarding fever (*P* = 0.01), fewer would provide antibiotics (*P* = 0.02) and more would provide traditional medicines (*P* = 0.03). Fewer would sell cefalexin without a prescription (*P* = 0.02) No statistically significant difference in reported change in steroid sales without a prescription (*P* = 0.12)
Phuong et al [38]	Randomised controlled trial	Bundle	Training on infectious diseases + provision of rapid diagnostic tests + combination of training and rapid diagnostic tests + control	48 months	Vietnam	2010	Outpatient: 12 primary health centres	Public	Presumptive and confirmed diagnoses Treatment of febrile patients Costs	Frequency of undifferentiated fever as a presumptive diagnosis increased in the infectious disease training intervention arm (OR = 2.103, *P* < 0.001) and provision of rapid diagnostic tests intervention arm (OR = 1.3888, *P* = 0.048). Antibiotic prescriptions increased dramatically in the control arm (OR = 10.334, *P* < 0.001), but declined in the intervention arm that provided rapid diagnostic tests (OR = 0.657, *P* = 0.002) and combination of training and provision of rapid diagnostic test arm (OR = 0.106, *P* < 0.001)

None of the included studies examined purely persuasive interventions, although similar components were included as part of a bundle intervention in nine studies [26, 28, 30, 32, 34, 35, 36, 37, 38]. Two studies were classified as enabling interventions that relied on continued medical education, other forms of education or guidelines for antibiotic use [27, 29]. Two studies by Rambaud‐Althaus et al and Shao et al utilised a new clinical algorithm for managing childhood illness and were hence classified as structural interventions: the former was a cluster randomised controlled trial conducted in programmatic conditions, while the latter was a controlled, non‐inferiority controlled trial [31, 33].

### Enabling interventions

All studies included in the review reported positive effects of the interventions through either increased knowledge around antibiotic use or reductions in inappropriate antibiotic prescriptions, with the exception of Das et al. This study found that informal providers (*n* = 304) who underwent 150 teaching hours over a 9‐month period did not report any significant change in antibiotic prescriptions [27]. Mean attendance at the 72 training sessions was 56% with no overlap from the control group, and the study found that informal providers in the intervention and control group prescribed 28.2% fewer antibiotics than public sector providers and improved correct case management rates (7.6% improvement; *P* < 0.05), despite no effect of the training programme on the use of unnecessary medicines and antibiotics. This study highlighted that interacting with and training informal providers who operate outside the purview of the formal medical system did not lead to any increase in violation of rules or clinical practice, which has been a common critique and misconception among medical doctors in India according to Das et al.

The only other study classified as an enabling intervention in our systematic review was Hoa et al’s two‐armed cluster randomised controlled trial with 304 medical and pharmacy staff in a rural district in Vietnam [29]. The interventions were conducted over seven months and included education around guidelines for appropriate antibiotic use, case scenario discussions and poster distribution. The authors examined both knowledge about the appropriateness of antibiotic use and practical clinical competence for management of children under five with acute respiratory infections (ARIs). The intervention group reported a 28% improvement in knowledge of ARI aetiology, and the mean improvement of 1.17 points in the total knowledge score within the intervention group was statistically significant (*P* < 0.001). When presented with a mild ARI case scenario, practical competence (reduction in use of antibiotics for ARIs) improved by over 20% in the intervention arm. However, there was no long‐term follow‐up to assess whether the change in knowledge was sustained over time and the difference in practical competence was only statistically significant (adjusted OR: 30.113; *P* = 0.048) for severe ARIs and not mild ARIs (adjusted OR: 1.003; *P* = 0.994).

### Structural interventions

Shao et al and Rambaud‐Althaus et al utilised a new electronic algorithm (ALMANACH) for the management of childhood illness using mobile phones in Tanzania. Shao et al tested the algorithm in controlled settings with 842 participants in the intervention arm and 623 participants in the control arm, and found that the use of the new algorithm achieved a better cure rate and a significantly lower rate of antibiotic prescription than routine practice (15.4% in the intervention arm vs. 84% in the control group; *P* < 0.001) [33]. Rambaud‐Althaus et al tested the same algorithm in programmatic conditions by randomising nine primary healthcare facilities into three arms with 504 children in total: paper algorithm (*n* = 171), electronic algorithm using a smartphone (*n* = 167) and a control group (*n* = 166). The study found that inappropriate antibiotic prescriptions dropped from 70% in the control arm to 26% in the paper algorithm and 25% in the electronic arm (*P* < 0.001) [31].

### Bundle interventions

The majority of studies (*n* = 9) within our systematic review were comprised of bundle interventions, which are multi‐faceted interventions integrating elements of an educational or training component along with either a persuasive or structural intervention. Kleczka et al integrated rubber stamp templates for management of pre‐specified medical conditions with clinical practice guidelines, one continued medical education session per month and a low‐budget smartphone to create a bundled intervention for 889 patient encounters at nine private sector health facilities in the informal sector. The study found that feedback did not affect the number of antibiotics prescribed for UTIs, but increased the use of appropriate, narrow‐spectrum antibiotics from 9.2% to 29.9% (*P* < 0.0001) and reduced the use of broad‐spectrum quinolones from 30% to 16.1% (*P* < 0.05) [26].

Two studies from Sudan, Awad et al and Eltayeb et al, utilised a pre‐post‐design in 20 health centres (*n* = 1800) to determine the impact of different multi‐faceted interventions: (1) no intervention, (2) graphs and explanations for audits of prescribing patterns, (3) audit and feedback along with seminars and a copy of guidelines, and (4) audit and feedback plus academic detailing along with a copy of guidelines [32, 34]. The former study focused on prescription patterns for all diseases at one and three‐month post‐intervention, while the latter focused specifically on sexually transmitted infections alone. The most effective intervention in both cases was the bundle intervention which combined audit and feedback with academic detailing through face‐to‐face educational meetings with prescribing experts and a copy of prescribing guidelines. Awad et al reported that audit and feedback in combination with academic detailing reduced the mean number of encounters with antibiotic prescriptions by 6.3 and 7.7 (*P* < 0.001) at 1‐month and 3‐months post‐intervention, respectively. Eltayeb et al reported a reduction of inappropriate prescriptions by 50% (*P* < 0.001) for this particular intervention, in comparison to 16% (*P* = 0.127) for audit and feedback alone.

Four studies focused on bundle interventions in Southeast Asia, specifically Vietnam and Thailand [35, 36, 37, 38]. Phuong et al conducted a randomised controlled trial with 60 primary health staff and 14,512 patients at 12 PHCs. Patients with acute undifferentiated fever (AUF) were recruited and PHCs were randomised to four intervention arms: (A) training on infectious diseases, (B) provision of rapid diagnostic tests (RDTs), (AB) combination of RDTs and training on infectious diseases or (C) control [38]. Prescriptions for antibiotics increased significantly during intervention in the control group (OR: 10.334, *P* < 0.001) and slightly, not significantly, in group A in comparison to the pre‐intervention period. Antibiotic prescriptions declined significantly in groups B (OR: 0.657, *P* = 0.002) and AB (OR: 0.106, *P* < 0.001).

Chuc et al (2002) and Chalker et al (2002) both examined the effect of multi‐faceted interventions on inappropriate antibiotic prescriptions as part of private pharmacy practice [36, 37]. Chalker et al conducted a randomised controlled trial with 22 matched pair intervention and control private pharmacies using the following interventions: (1) regulation enforcement, (2) face‐to‐face education and (3) peer influence. The study was one of the first to conduct a multi‐intervention study in the private pharmacy sector in a low‐income country. While there was no significant change in selling steroids without a prescription between intervention and control pharmacies (*P* = 012), there was a significant decline (56.8% to 20%; *P* = 0.02) in the number of intervention pharmacies that would sell antibiotics without a prescription. ???? and the control group increased from 45% to 61% (*P* = 0.02) [37]. Similarly, Chuc et al used four tracer conditions (uncomplicated acute respiratory infections, sexually transmitted disease, requests for the prescription‐only drugs prednisolone and a short course of cephalexin) in 68 randomly selected pharmacies in Hanoi and found that dispensing of antibiotics decreased in intervention pharmacies from 45% to 30% (*P* < 0.02) and increased in control pharmacies from 39% to 42% (*P* < 0.05) [36]. Both studies espoused the value of multi‐pronged interventions as an effective way to curb antibiotic misuse in private pharmacy practice. Another study by Chalker et al from 2005 examined the effectiveness of a similar multi‐faceted intervention in 68 Hanoi pharmacies and 78 Bangkok pharmacies [35]. Three interventions (regulation enforcement with local inspectors, face‐to‐face education in Hanoi and large‐group education in Bangkok; and voluntary peer review in Bangkok with compulsory peer review in Hanoi) were implemented sequentially with four months in between and the interventions resulted in significant improvements in Hanoi, but not Bangkok [35]. Dispensing of antibiotics improved in Hanoi after peer review intervention from 95% to 71% (*P* = 0.0125) and showed a smaller improvement post educational intervention from 90% to 69% (*P* = 0.0471), but there was no statistically significant difference between intervention and control arms in Bangkok (*P* > 0.05).

Korom et al conducted a pre‐post effectiveness trial in Kenya, which examined the effect of brief educational interventions, including (1) a clinical practice guideline, (2) peer‐to‐peer chart review and (3) peer‐reviewed literature detailing local AMR patterns, on the proportion of cases (*n* = 474) in which appropriate antibiotics were prescribed as per the guidelines [30]. Clinical adherence improved from 19% to 68% after all interventions (*P* < 0.001) [30]. Finally, Lunn measured the effect of a multi‐faceted intervention (*n* = 222) that involved audit and prescribing feedback, educational seminars, one‐on‐one case‐based discussions, guideline development and a change in the way the disease is medically coded on reducing inappropriate antibiotic prescriptions in India [28]. The study reported a reduction in antibiotic prescriptions from 62.6% to 7.2% and increased documentation of examination findings (52.7% to 95.6%). This intervention was particularly salient because it relied on low‐cost resources in an NGO’s outreach clinics in India [28]. However, the author did not provide any evidence of statistical significance despite claims to the contrary and *P*‐values are missing from the review.

### Risk of bias

In general, there was a fairly high risk of bias across most studies in the review, especially given the inclusion of non‐randomised studies (see Table 2 below).

**Table 2:** Risk of bias across studies

Authors	Type of study	Selection bias (random sequence generation)	Selection bias (allocation concealment)	Reporting bias (selective reporting)	Performance bias (blinding of participants and personnel)	Detection bias (blinding outcome assessment)	Attrition bias (incomplete outcome data)
Kleczka et al [26]	Pre‐post design	High	High	High	High	High	High
Das et al [27]	Randomised controlled trial	Low	Low	Low	Low	Unclear	Low
Lunn AD [28]	Pre‐post‐design	High	High	High	High	High	Low
Hoa et al [29]	Randomised controlled trial	Low	Low	Low	High	Unclear	Unclear
Korom et al [30]	Pre‐post‐design	High	High	High	High	High	Low
Rambaud‐Althaus et al [31]	Cluster randomised controlled trial	Low	Low	Low	High	High	Low
Eltayeb et al [32]	Pre‐post design	High	Low	Low	High	High	Low
Shao et al [33]	Controlled, non‐inferiority trial	Low	Low	Low	High	High	Low
Awad et al [34]	Pre‐post design	High	High	Low	High	High	Low
Chalker et al [35]	Randomised controlled trial	Low	Low	Low	High	High	Low
Chuc et al [36]	Cluster randomised controlled trial with time series design	Low	Low	Low	High	High	Low
Chalker et al [37]	Randomised controlled trial	Low	Low	Low	High	High	Low
Phuong et al [38]	Randomised controlled trial	Low	High	Low	High	High	Unclear

### Other observations

Response shift bias is a common source of bias among non‐randomised study designs (Kleczka et al, Lunn, Korom et al, Eltayeb et al, and Awad et al). In the study by Das et al, while the risk of bias according to traditional criteria was quite low, there was an additional risk because the impact of training was only estimated for those who expressed interest in the programme, rather than all participants who were approached. Similarly, with Hoa et al’s randomised controlled trial, there was a difference in participation between private and public facilities which may have biased results; Korom et al’s pre‐post study only covered two outpatient sites and relied on a retrospective chart review to assess process measures in clinical encounters, which limits generalisability and discounts the possibility of assessments without adequate documentation. The average interval between intervention and outcome measurement for studies was 3.5 months, and the intervention follow‐up period varied dramatically from 28 days (Shao et al [33]) to 5 years (Phuong et al [38]). In general, the durability of the interventions is unclear, because most studies did not assess the long‐term impact of the intervention beyond 12 months.

## Discussion

In general, educational interventions alone tend to have smaller effect sizes than multi‐faceted interventions that combine education with other components such as audit and feedback, provision of rapid diagnostic tests or academic detailing [32, 33, 34, 35, 36, 37, 38]. For instance, evidence from randomised controlled trials conducted by Chalker et al and Chuc et al demonstrates the importance of moving beyond educational interventions to include multi‐faceted bundle interventions that combine regulation enforcement with face‐to‐face education and peer influence. Based on the 13 studies included in our final review, bundle interventions appear to be effective at changing prescription behaviour among healthcare providers, including drug sellers and pharmacists. However, many studies included in our review had a high risk of bias given the pre‐post design and the lack of randomisation. Further evidence from rigorously conducted randomised controlled trials is required to test the efficacy of these interventions, particularly among pharmacists, drug sellers and other informal providers. We also found more interventions in the private sector in comparison to the public sector in our review, which may be due to the bureaucratic difficulties in conducting and publishing interventions within public facilities in LMICs.

Our systematic review features studies from outpatient settings in LMICs, with several studies focusing on the informal sector and pharmaceutical sector which is underrepresented in the literature on ABR from these regions. The study by Das et al is critical in illustrating the importance of improving the evidence base for behavioural interventions in the informal sector, which caters to 50‐80% of primary healthcare visits in rural India and represents a major driver of antibiotic use in India [39, 40, 41]. While the educational intervention did not result in significant changes to rational antibiotic prescription behaviour, it did improve correct case management, suggesting the informal sector can be reached for behavioural interventions, without cause for additional concern around worsening existing practices. Das et al indicate that the poor effect of the intervention on antibiotic prescriptions may result from the fact that the majority of providers earn profits through sales of medicines from wholesale providers or sales representatives, which may incentivise overtreatment.

Arnold and Strauss reported similar results in their systematic review, which analysed 39 studies detailing the effect of ‘printed educational materials for physicians, audit and feedback, educational meetings, educational outreach visits, financial and healthcare system changes, physician reminders, patient‐based interventions and multi‐faceted interventions’ in high‐income contexts [22]. Their results suggest that printed educational materials or audit and feedback alone result in little or no change in prescription behaviour and multi‐faceted or bundle interventions are the most effective in reducing irrational antibiotic use [22]. Other studies have also found that simply raising the awareness of ABR among consumers or prescribers in LMICs may not be effective, because prescribers weigh a multitude of options when prescribing antibiotics: Pearson and Chandler found that high levels of awareness in nine low‐income settings did not translate into a reduction in misuse of antibiotics [42]. Prescription behaviour was shaped by social determinants, such as the prevailing economic situation, decrepit health infrastructure and inadequate hygiene and subsequent reliance on antibiotics as a substitute.

In resource‐limited contexts within countries like India or Nigeria, successful and sustainable interventions should incorporate elements of audit and feedback or academic detailing interventions. Given the difficulties of implementing academic detailing interventions in resource‐limited contexts; however, reinforcement seminars may be an effective alternative as suggested by Awad et al [34]. There is a need to evaluate the durability and sustainability of these interventions long‐term, especially in limited‐resource settings, but multi‐faceted interventions have a better track record to influence prescription behaviour in this regard, compared to any single intervention. As Kleczka et al found, the use of routine data to measure and improve quality of healthcare is rare in LMICs and simple technological interventions, such as rubber stamp templates and the use of smartphones, to deliver routine data in outpatient settings may also be an effective complement to educational or audit and feedback interventions in order to address knowledge–practice gaps within the health system.

Regulatory enforcement is often a major challenge in low‐income settings and may cause more harm than good: as Goodman et al note, overzealous regulatory enforcement may have the unintended consequence of limiting access to urgently needed medicines, including antibiotics [43]. Rather than envisioning a top‐down paradigm of regulatory enforcement, one possible way forward is to leverage multi‐sectoral coordination and utilise a combination of provider incentives, indirect regulation through consumers and public–private collaboration to incentivise rational prescription and dispensation of antibiotics [43, 44]. In the Indian context for example, a potential way forward would be to link existing behavioural interventions or educational seminars with organised associations of informal providers and pharmacists, who function as the frontline providers in many rural areas. Adopting appropriate or rational prescription behaviour requires a shift away from reliance on guidelines or educational interventions alone, and a broader recognition of the systemic factors driving prescription decisions, such as the use of antibiotics as a way to prevent future infections in the context of poor sanitation and infection control [41]. Structural interventions alone may offer some benefit over educational interventions, but they will be more effective and sustainable in concert with audit and feedback and other behavioural interventions that are integrated within the health system in limited‐resource settings.

### Limitations

Given the heterogeneity of the studies, we were unable to conduct a meta‐analysis and decided to opt for a narrative synthesis of the studies. We excluded national policy guidelines as they were determined to be outside the scope of behavioural interventions, and we did not include studies that focused simply on cost‐effectiveness. Our review focused solely on prescription behaviour and did not take into account behavioural interventions targeting consumers or the general public – this has been adequately addressed by other reviews in high‐income countries, but remains an area with insufficient scientific literature in LMICs. Most of the studies included in the review measured changes over a short period of time and did not adequately account for long‐term or sustainable changes in behaviour. There was also a high risk of bias among the pre‐post studies included in the review. Our review primarily focused on three major databases (PubMed, Embase and CENTRAL), and did not comprehensively cover grey literature due to resource limitations. The generalisability of this review may be limited given the scope, but there are important lessons to be gleaned from the varied interventions in diverse low‐income and lower‐middle‐income countries included in the review.

## Conclusion

Multi‐faceted interventions that integrate educational materials with audit and feedback or peer‐to‐peer comparison may be a more effective method of reducing inappropriate prescriptions in limited‐resource, outpatient settings than educational interventions alone, but there is not enough high‐quality evidence from randomised controlled trials and further research is required to test the sustainability of these interventions.

## Supporting information


**Appendix S1.** PRISMA checklist
**Appendix S2.** Study protocol and search strategy
