# Supplementary material for: Behavioural interventions to address rational use of antibiotics in outpatient settings of low‐income and lower‐middle‐income countries
Source: Trop Med Int Health. 2021 Jan 28;26(5):504–17. doi: 10.1111/tmi.13550 (PMC8248115; doi:10.1111/tmi.13550)
Supplement: Supplementary file 1 — Appendix S1. PRISMA checklist Appendix S2. Study protocol and search strategy [file TMI-26-504-s001.docx]

**Appendix 1:** PRISMA checklist

| **Section/topic** | **#** | **Checklist item** | **Reported on page #** |
| --- | --- | --- | --- |
| **TITLE** | | |  |
| Title | 1 | Identify the report as a systematic review, meta-analysis or both. | 1 |
| **ABSTRACT** | | |  |
| Structured summary | 2 | Provide a structured summary including, as applicable: background; objectives; data sources; study eligibility criteria, participants and interventions; study appraisal and synthesis methods; results; limitations; conclusions and implications of key findings; systematic review registration number. | 2 |
| **INTRODUCTION** | | |  |
| Rationale | 3 | Describe the rationale for the review in the context of what is already known. | 4-5 |
| Objectives | 4 | Provide an explicit statement of questions being addressed with reference to participants, interventions, comparisons, outcomes and study design (PICOS). | 4-5 |
| **METHODS** | | |  |
| Protocol and registration | 5 | Indicate if a review protocol exists, if and where it can be accessed (e.g. Web address), and, if available, provide registration information including registration number. | 5 |
| Eligibility criteria | 6 | Specify study characteristics (e.g. PICOS and length of follow-up) and report characteristics (e.g. years considered, language and publication status) used as criteria for eligibility, giving rationale. | 5-6 |
| Information sources | 7 | Describe all information sources (e.g. databases with dates of coverage and contact with study authors to identify additional studies) in the search and date last searched. | 5-9 |
| Search | 8 | Present full electronic search strategy for at least one database, including any limits used, such that it could be repeated. | Supplementary file/Appendix |
| Study selection | 9 | State the process for selecting studies (i.e. screening, eligibility, included in systematic review and, if applicable, included in the meta-analysis). | 9 |
| Data collection process | 10 | Describe method of data extraction from reports (e.g. piloted forms, independently and in duplicate) and any processes for obtaining and confirming data from investigators. | 9 |
| Data items | 11 | List and define all variables for which data were sought (e.g. PICOS and funding sources) and any assumptions and simplifications made. | 6 |
| Risk of bias in individual studies | 12 | Describe methods used for assessing risk of bias of individual studies (including specification of whether this was done at the study or outcome level), and how this information is to be used in any data synthesis. | 7 |
| Summary measures | 13 | State the principal summary measures (e.g. risk ratio and difference in means). | 9 |
| Synthesis of results | 14 | Describe the methods of handling data and combining results of studies, if done, including measures of consistency (e.g. I^2^) for each meta-analysis. | N/A |

Page 1 of 2

| **Section/topic** | **#** | **Checklist item** | **Reported on page #** |
| --- | --- | --- | --- |
| Risk of bias across studies | 15 | Specify any assessment of risk of bias that may affect the cumulative evidence (e.g. publication bias and selective reporting within studies). | 7 |
| Additional analyses | 16 | Describe methods of additional analyses (e.g. sensitivity or subgroup analyses, and meta-regression), if done, indicating which were pre-specified. | N/A |
| **RESULTS** | | |  |
| Study selection | 17 | Give numbers of studies screened, assessed for eligibility and included in the review, with reasons for exclusions at each stage, ideally with a flow diagram. | 8 |
| Study characteristics | 18 | For each study, present characteristics for which data were extracted (e.g. study size, PICOS and follow-up period) and provide the citations. | 10-30 |
| Risk of bias within studies | 19 | Present data on risk of bias of each study and, if available, any outcome level assessment (see item 12). | 36-39 |
| Results of individual studies | 20 | For all outcomes considered (benefits or harms), present, for each study: (a) simple summary data for each intervention group (b) effect estimates and confidence intervals, ideally with a forest plot. | 10-30 |
| Synthesis of results | 21 | Present results of each meta-analysis done, including confidence intervals and measures of consistency. | N/A |
| Risk of bias across studies | 22 | Present results of any assessment of risk of bias across studies (see Item 15). | 36-39 |
| Additional analysis | 23 | Give results of additional analyses, if done (e.g. sensitivity or subgroup analyses, and meta-regression [see Item 16]). | N/A |
| **DISCUSSION** | | |  |
| Summary of evidence | 24 | Summarise the main findings including the strength of evidence for each main outcome; consider their relevance to key groups (e.g. healthcare providers, users and policy makers). | 40-41 |
| Limitations | 25 | Discuss limitations at study and outcome level (e.g. risk of bias), and at review-level (e.g. incomplete retrieval of identified research and reporting bias). | 44 |
| Conclusions | 26 | Provide a general interpretation of the results in the context of other evidence and implications for future research. | 44 |
| **FUNDING** | | |  |
| Funding | 27 | Describe sources of funding for the systematic review and other support (e.g. supply of data); role of funders for the systematic review. | 46 |

*From:*  Moher D, Liberati A, Tetzlaff J, Altman DG, The PRISMA Group (2009). Preferred Reporting Items for Systematic Reviews and Meta-Analyses: The PRISMA Statement. PLoS Med 6(7): e1000097. doi:10.1371/journal.pmed1000097

For more information, visit: **www.prisma-statement.org**.

**Appendix 2:** Study protocol and search strategy

**Background:** Global antibiotic consumption, expressed in defined daily doses (DDDs), increased by 65% from 21.1 billion DDDs to 34.8 billion DDDs between 2000 and 2015, largely driven by rising consumption in low- and middle-income countries, according to a recent study published in Proceedings of the National Academy of Sciences [1]. Given the burden of infectious diseases in LMICs, irrational use of antibiotics is a common practice and compounded further by self-medication and over-the-counter use among patients, which are well-established drivers of antibiotic resistance (ABR) [2-6]. Despite this evidence however, the literature on behavioural interventions, such as randomised control trials analysing peer comparison feedback mechanisms or accountable justification, to curb irrational use of antibiotics is very limited [7-9]. Where studies exist, the vast majority take place in OECD countries which do not face the same burdens or drivers as LMICs.

# There are several systematic reviews which look at drivers of antibiotic use or resistance rates based on antimicrobial susceptibility testing, and this is well-documented in the ABR literature [10-17]. However, there were no systematic reviews which focused on behavioural interventions to control antibiotic resistance, in LMICs or otherwise. Given this evidence gap and the WHO commitment to tackle antibiotic resistance before it becomes the leading cause of mortality by 2050, this study will aim to conduct a systematic review of behavioural interventions to address ABR in LMICs.

# Objective: To estimate the effectiveness of behavioural interventions to improve antibiotic prescribing in lower and middle-income countries.

# PICO framework

| **Population** | Healthcare providers (doctors, informal doctors, nurses, pharmacists, CHWs) in LMICs who prescribe or provide antibiotics in primary care, outpatient or community-based settings |
| --- | --- |
| **Problem** | Irrational or unnecessary overuse of antibiotics |
| **Intervention** | Any behavioural intervention targeted towards healthcare professionals to reduce irrational antibiotic prescriptions and improve antibiotic use. |
| **Comparison** | Usual care or standard practice (no intervention to influence prescription behaviour) |
| **Outcome** | Primary outcome: reduction in irrational or unnecessary antibiotic use (i.e. use of antibiotics for viral infections or improved adherence to guidelines). Secondary outcome: improvement in KAP around antibiotic use  Rational antibiotic use is defined as the right antibiotic at the right dose for the right duration at the right time, as per the CDC definition. |

# Search Strategy

| **Databases**  *List the bibliographic databases to be searched.* |
| --- |
| We will search PubMed, Embase and Cochrane Central Register of Controlled Trials for studies relating to antibiotic prescribing and ambulatory care. We will also examine the bibliographies of retrieved articles. Only articles which were published in English between 2001 and 2020 will be included in the review. Articles published before the year 2001 will be excluded, as the first global plans to address the problem of antibiotic resistance emerged that year [18]. |

Afghanistan[tw] OR Albania[tw] OR Algeria[tw] OR "American Samoa"[tw] OR Angola[tw] OR Argentina[tw] OR "Argentine

Republic"[tw] OR Armenia[tw] OR Azerbaijan[tw] OR Bangladesh[tw] OR Belarus[tw] OR Byelarus[tw] OR Belorussia[tw] OR Belize[tw]

OR Benin[tw] OR Bhutan[tw] OR Bolivia[tw] OR Bosnia[tw] OR Botswana[tw] OR Brazil[tw] OR Bulgaria[tw] OR Burma[tw] OR

"Burkina Faso"[tw] OR Burundi[tw] OR "Cabo Verde"[tw] OR "Cape verde"[tw] OR Cambodia[tw] OR Cameroon[tw] OR "Central

African Republic"[tw] OR Chad[tw] OR China[tw] OR Colombia[tw] OR Comoros[tw] OR Comores[tw] OR Comoro[tw] OR Congo[tw]

OR "Costa Rica"[tw] OR "Cote d'Ivoire"[tw] OR Cuba[tw] OR Djibouti[tw] OR Dominica[tw] OR "Dominican Republic"[tw] OR

Ecuador[tw] OR Egypt[tw] OR "El Salvador"[tw] OR Eritrea[tw] OR Ethiopia[tw] OR Fiji[tw] OR Gabon[tw] OR Gambia[tw] OR

Gaza[tw] OR "Georgia Republic"[tw] OR Georgian[tw] OR Ghana[tw] OR Grenada[tw] OR Grenadines[tw] OR Guatemala[tw] OR

Guinea[tw] OR "Guinea Bissau"[tw] OR Guyana[tw] OR Haiti[tw] OR Herzegovina[tw] OR Hercegovina[tw] OR Honduras[tw] OR

India[tw] OR Indonesia[tw] OR Iran[tw] OR Iraq[tw] OR Jamaica[tw] OR Jordan[tw] OR Kazakhstan[tw] OR Kenya[tw] OR Kiribati[tw]

OR Korea[tw] OR Kosovo[tw] OR Kyrgyz[tw] OR Kirghizia[tw] OR Kirghiz[tw] OR Kirgizstan[tw] OR Kyrgyzstan[tw] OR "Lao

PDR"[tw] OR Laos[tw] OR Lebanon[tw] OR Lesotho[tw] OR Liberia[tw] OR Libya[tw] OR Macedonia[tw] OR Madagascar[tw] OR

Malawi[tw] OR Malay[tw] OR Malaya[tw] OR Malaysia[tw] OR Maldives[tw] OR Mali[tw] OR "Marshall Islands"[tw] OR Mauritania[tw]

OR Mauritius[tw] OR Mexico[tw] OR Micronesia[tw] OR Moldova[tw] OR Mongolia[tw] OR Montenegro[tw] OR Morocco[tw] OR

Mozambique[tw] OR Myanmar[tw] OR Namibia[tw] OR Nauru[tw] OR Nepal[tw] OR Nicaragua[tw] OR Niger[tw] OR Nigeria[tw] OR

Pakistan[tw] OR Palau[tw] OR Panama[tw] OR "Papua New Guinea"[tw] OR Paraguay[tw] OR Peru[tw] OR Philippines[tw] OR

Phillippines[tw] OR Philipines[tw] OR Phillipines[tw] OR Principe[tw] OR Romania[tw] OR Rwanda[tw] OR Ruanda[tw] OR Samoa[tw]

OR "Sao Tome"[tw] OR Senegal[tw] OR Serbia[tw] OR "Sierra Leone"[tw] OR "Solomon Islands"[tw] OR Somalia[tw] OR "South

Africa"[tw] OR "South Sudan"[tw] OR "Sri Lanka"[tw] OR "St Lucia"[tw] OR "St Vincent"[tw] OR Sudan[tw] OR Surinam[tw] OR

Suriname[tw] OR Swaziland[tw] OR Syria[tw] OR "Syrian Arab Republic"[tw] OR Tajikistan[tw] OR Tadzhikistan[tw] OR Tadjikistan[tw]

OR Tadzhik[tw] OR Tanzania[tw] OR Thailand[tw] OR Timor[tw] OR Togo[tw] OR Tonga[tw] OR Tunisia[tw] OR Turkey[tw] OR

Turkmen[tw] OR Turkmenistan[tw] OR Tuvalu[tw] OR Uganda[tw] OR Ukraine[tw] OR Uzbek[tw] OR Uzbekistan[tw] OR Vanuatu[tw]

OR Venezuela[tw] OR Vietnam[tw] OR "West Bank"[tw] OR Yemen[tw] OR Zambia[tw] OR Zimbabwe[tw] AND "Anti-Bacterial

Agents"[Mesh] OR "Anti-Bacterial Agents"[Pharmacological Action] OR "Penicillins"[Mesh] OR (antibacterial[tiab] OR antibacterial'[tiab]

OR antibacterial's[tiab] OR antibacterialactivity[tiab] OR antibacterialeffects[tiab] OR antibacteriall[tiab] OR antibacterially[tiab] OR

antibacterials[tiab] OR antibacterials'[tiab]) OR (anti bacterial[tiab] OR anti bacterially[tiab] OR anti bacterials[tiab]) OR (antibiotic[tiab] OR

antibiotic'[tiab] OR antibiotic'ome[tiab] OR antibiotic's[tiab] OR antibiotic120[tiab] OR antibiotic2[tiab] OR antibiotica[tiab] OR

antibioticabeleid[tiab] OR antibioticabeleid'[tiab] OR antibiotical[tiab] OR antibioticalis[tiab] OR antibiotically[tiab] OR antibioticand[tiab]

OR antibioticas[tiab] OR antibioticassociated[tiab] OR antibioticbacterial[tiab] OR antibioticchoice[tiab] OR antibioticdb[tiab] OR

antibiotice[tiab] OR antibiotices[tiab] OR antibioticfor[tiab] OR antibioticfuture[tiab] OR antibioticgram[tiab] OR antibioticguardian[tiab]

OR antibiotiche[tiab] OR antibiotichordecin[tiab] OR antibiotichyaluronidase[tiab] OR antibiotici[tiab] OR antibioticimpregnated[tiab] OR

antibioticinduced[tiab] OR antibioticl[tiab] OR antibioticlike[tiab] OR antibioticloaded[tiab] OR antibiotico[tiab] OR antibioticogram[tiab] OR antibioticogrammes[tiab] OR antibioticogramms[tiab] OR antibioticograms[tiab] OR antibioticolade[tiab] OR antibioticone[tiab] OR

antibioticophila[tiab] OR antibioticoprofilaxia[tiab] OR antibioticoprophylaxis[tiab] OR antibioticoresistance[tiab] OR

antibioticoresistant[tiab] OR antibioticoresistent[tiab] OR antibioticos[tiab] OR antibioticosensetivity[tiab] OR antibioticosensitivity[tiab]

OR antibioticoterapia[tiab] OR antibioticoterapy[tiab] OR antibioticotherapies[tiab] OR antibioticotherapy[tiab] OR

antibioticotropism[tiab] OR antibioticprobiotic[tiab] OR antibioticprophilaxy[tiab] OR antibioticreleasing[tiab] OR antibioticresistance[tiab]

OR antibioticresistant[tiab] OR antibioticreviewkit[tiab] OR antibiotics[tiab] OR antibiotics'[tiab] OR antibiotics''[tiab] OR

antibiotics`effect[tiab] OR antibioticsalone[tiab] OR antibioticsantibiotic[tiab] OR antibioticscompared[tiab] OR antibioticscout[tiab] OR

antibioticsdrug[tiab] OR antibioticsdue[tiab] OR antibioticsendocardiectomyimplantable[tiab] OR antibioticsfor[tiab] OR antibioticsin[tiab]

OR antibioticsis[tiab] OR antibioticsl[tiab] OR antibioticsmore[tiab] OR antibioticsolder[tiab] OR antibioticsone[tiab] OR

antibioticstewardship[tiab] OR antibioticsts[tiab] OR antibioticsubstances[tiab] OR antibioticsusceptible[tiab] OR antibiotictherapy[tiab]

OR antibiotictreated[tiab] OR antibiotictreatment[tiab] OR antibioticum[tiab] OR antibioticunresponsive[tiab] OR antibioticus[tiab] OR

antibioticustu[tiab] OR antibioticwe[tiab] OR antibioticwhen[tiab] OR antibioticxuebijing[tiab]) OR (amoxicilline[tiab] OR

amoxicillines[tiab]) OR flucloxacilline[tiab] OR piperacilline[tiab] AND "Prescriptions"[Mesh] OR "Prescription Drug Misuse"[Mesh] OR

"Prescription Drugs"[Mesh] OR "Inappropriate Prescribing"[Mesh] AND intervention [tiab]

# Eligibility Criteria

| **Inclusion Criteria** | **Exclusion Criteria** |
| --- | --- |
| - Evaluation of an intervention that influences antibiotic prescription behaviour (i.e. education, restriction and accountable justification) - Population includes healthcare providers in the primary care, outpatient or other community-based setting - Study assessed an outcome measure related to human antimicrobial use such as dosing or prescribing - Randomised controlled trials, non-randomised controlled trials, time series design, non-randomised controlled before-and-after design, uncontrolled before-and-after design, observational studies, qualitative studies | - Studies not pertaining to human antimicrobial use - Studies focusing on hospital inpatients or long-term nursing care patients - Studies which are not conducted in an LMIC - Studies that only reported cost-effectiveness outcomes - Studies that looked at infection prevention and control rather than prescription interventions - Studies looking at the impact of national or hospital-based policies - Studies with no full-text articles available - Studies looking at microbiological testing - Studies published before 2001 will be excluded |

# Selection of Studies and Data Extraction

One review researcher will review all citations and abstracts to identify interventions addressing antibiotic prescription behaviour, according to the inclusion and exclusion criteria. The list of included studies will be determined independently first and reviewed by senior authors. The reviewer will use a pre-designed data extraction sheet including information on study design, type of intervention, type of targeted behaviour, participants, setting, methods, outcomes and results.

# References

1. Klein EY, Van Boeckel TP, Martinez EM, Pant S, Gandra S, Levin SA, et al. Global increase and geographic convergence in antibiotic consumption between 2000 and 2015. Proceedings of the National Academy of Sciences. 2018 Mar 21:201717295.
2. Abdulah R (2012) Antibiotic Abuse in Developing Countries. Pharmaceut Reg Affairs 1:e106. doi:10.4172/2167-7689.1000e106
3. Orrett FA (2001)[Antimicrobial prescribing patterns at a rural hospital in Trinidad: evidence for intervention measures. Afr J Med Med Sci 30: 161-164.](https://www.ncbi.nlm.nih.gov/pubmed/14510121)
4. Ansari F (2001)[Use of systemic anti-infective agents in Iran during 1997–1998. Eur J Clin Pharmacol 57: 547-551.](https://www.ncbi.nlm.nih.gov/pubmed/11699624)
5. Chukwuani CM, Onifade M, Sumonu K (2002)[Survey of drug use practices and antibiotic prescribing at a general hospital in Nigeria. Pharm World Sci 24: 188-195.](https://www.ncbi.nlm.nih.gov/pubmed/12426963)
6. Arya SC (2004)[Antibiotics prescription in hospitalized patients at a Chinese university hospital. J Infect 48: 117-118.](https://www.ncbi.nlm.nih.gov/pubmed/14667802)
7. Linder JA, Meeker D, Fox CR, Friedberg MW, Persell SD, Goldstein NJ, Doctor JN. Effects of behavioral interventions on inappropriate antibiotic prescribing in primary care 12 months after stopping interventions. Jama. 2017 Oct 10;318(14):1391-2.
8. Meeker D, Linder JA, Fox CR, Friedberg MW, Persell SD, Goldstein NJ, Knight TK, Hay JW, Doctor JN. Effect of behavioral interventions on inappropriate antibiotic prescribing among primary care practices: a randomized clinical trial. Jama 2016 Feb 9;315(6):562-70.
9. Chatterjee A, Modarai M, Naylor NR, Boyd SE, Atun R, Barlow J, Holmes AH, Johnson A, Robotham JV. Quantifying drivers of antibiotic resistance in humans: a systematic review. The Lancet Infectious Diseases. 2018 Aug 29.
10. Nellums LB, Thompson H, Holmes A, Castro-Sánchez E, Otter JA, Norredam M, Friedland JS, Hargreaves S. Antimicrobial resistance among migrants in Europe: a systematic review and meta-analysis. The Lancet Infectious Diseases. 2018 Jul 1;18(7):796-811.
11. Bell BG, Schellevis F, Stobberingh E, Goossens H, Pringle M. A systematic review and meta-analysis of the effects of antibiotic consumption on antibiotic resistance. BMC infectious diseases. 2014 Dec;14(1):13.
12. Britto CD, John J, Verghese VP, Pollard AJ. A systematic review of antimicrobial resistance of typhoidal Salmonella in India. The Indian Journal of Medical Research. 2019 Feb;149(2):151.
13. Irek EO, Amupitan AA, Obadare TO, Aboderin AO. A systematic review of healthcare-associated infections in Africa: An antimicrobial resistance perspective. African journal of laboratory medicine. 2018;7(2):1-9.
14. Warremana EB, Lambregtsa MM, Wouters RH, Visser LG, Staats H, van Dijk E, de Boer MG. Determinants of in-hospital antibiotic prescription behaviour: a systematic review and formation of a comprehensive framework. Category: systematic review. Clinical Microbiology and Infection. 2018.
15. Muhie OA. Antibiotic Use and Resistance Pattern in Ethiopia: Systematic Review and Meta-Analysis. International Journal of Microbiology. 2019.
16. Persell SD, Doctor JN, Friedberg MW, Meeker D, Friesema E, Cooper A, Haryani A, Gregory DL, Fox CR, Goldstein NJ, Linder JA. Behavioral interventions to reduce inappropriate antibiotic prescribing: a randomized pilot trial. BMC infectious diseases. 2016 Dec;16(1):373.
17. Ahmed I, Rabbi MB, Sultana S. Antibiotic resistance in Bangladesh: A systematic review. International Journal of Infectious Diseases. 2019 Jan 10.
18. Rzewuska M, Duncan E, Marwick C, Ramsay C. A systematic review of barriers and enablers to implementation of antibiotic stewardship programs in inpatient hospital settings. PROSPERO 2017 CRD42017076425. Available from: <https://www.crd.york.ac.uk/prospero/display_record.php?ID=CRD42017076425>
